# Effect of a Videoconference-Based Online Group Intervention for Traumatic Stress in Parents of Children With Life-threatening Illness: A Randomized Clinical Trial

**DOI:** 10.1001/jamanetworkopen.2020.8507

**Published:** 2020-07-31

**Authors:** Frank Muscara, Maria C. McCarthy, Meredith Rayner, Jan M. Nicholson, Anica Dimovski, Laura McMillan, Stephen J. C. Hearps, Jackie Yamada, Kylie Burke, Robyn Walser, Vicki A. Anderson

**Affiliations:** 1 Clinical Sciences, Murdoch Children’s Research Institute, The Royal Children’s Hospital, Parkville, Victoria, Australia; 2 Children’s Cancer Centre, The Royal Children’s Hospital, Parkville, Victoria, Australia; 3 Judith Lumley Centre, La Trobe University, Bundoora, Victoria, Australia; 4 Parenting and Family Support Centre, School of Psychology, The University of Queensland, Brisbane, Queensland, Australia; 5 Department of Psychology, University of California, Berkeley; 6 TL Consultation Services, Menlo Park, California

## Abstract

**Importance:**

A substantial proportion of parents whose child is diagnosed with a life-threatening illness experience high levels of distress that can lead to long-term mental health difficulties. This can affect the child’s recovery.

**Objective:**

To evaluate the efficacy of an acceptance and commitment therapy–based group intervention, delivered using videoconferencing, in reducing posttraumatic stress symptoms (PTSS) in these parents.

**Design, Setting, and Participants:**

This study was a randomized clinical trial of an intervention for parents with elevated acute stress symptoms. It was a single-site study conducted in a tertiary pediatric hospital in Australia. Parents of children aged 0 to 18 years admitted for a life-threatening illness or injury to the oncology, cardiology, or pediatric intensive care departments were eligible. Participants were screened for eligibility within the first month after diagnosis or admission and then were randomized to the intervention group or the waiting list control group 4 to 10 months after diagnosis or admission. Recruitment commenced January 2014, and final postintervention follow-up was completed in February 2018. Data analysis was performed from July to September 2018.

**Interventions:**

Treatment was a psychological acceptance and commitment therapy–based group therapy program called Take a Breath, which consisted of a 6-session parent-mediated psychological intervention delivered via online videoconferences over the course of 8 weeks. Waiting list control participants received treatment as usual and were offered the intervention 3 months after randomization.

**Main Outcomes and Measures:**

The primary outcome was PTSS, as measured by the Posttraumatic Stress Disorder Checklist–Version 5 (total score range, 0-80, with higher scores indicating greater symptom severity). The PTSS was measured both before and immediately after the intervention. Changes in psychological skills taught within the intervention were also evaluated, including acceptance, mindfulness, values-based living, and psychological flexibility.

**Results:**

Of 1232 parents who were assessed for eligibility, 313 were randomized; 161 were allocated to the waiting list control group, and 152 were allocated to the intervention group. Of those allocated, 44 parents in the waiting list group and 37 in the intervention group completed the postintervention questionnaire and were analyzed (81 participants total; mean [SD] age, 37.17 [6.43] years). Sixty-five participants (80.2%) were women, 48 participants (59.3%) were married, and 40 participants (49.4%) lived in rural or regional areas, or in a different state. In addition, 24 parents (29.6%) were in the cardiology illness group, 32 parents (39.5%) were in the oncology group, and 25 parents (30.9%) were in the pediatric intensive care unit group. The intervention group demonstrated significantly greater improvements in PTSS compared with the waiting list group (Cohen *d* = 1.10; 95% CI, 0.61-1.59; *P* = .03). The mean Posttraumatic Stress Disorder Checklist–Version 5 scores decreased from 31.7 (95% CI, 27.0-36.4) to 26.2 (95% CI, 21.8-30.7) in the waiting list control group and from 23.3 (95% CI, 18.6-28.1) to 17.8 (95% CI, 13.8-21.8) in the intervention group.

**Conclusions and Relevance:**

The findings of this study support the use of acceptance and commitment therapy to reduce PTSS in parents of very ill children, regardless of diagnosis. These findings also suggest that a brief, group format using a videoconferencing platform can be used effectively to access hard-to-reach populations, particularly fathers and caregivers living in nonmetropolitan areas.

**Trial Registration:**

Australian New Zealand Clinical Trials Registry Identifier: ACTRN12611000090910

## Key Points

**Question:**

Is an acceptance and commitment therapy–based group intervention, delivered using videoconferencing, effective in reducing posttraumatic stress symptoms in parents of very ill children?

**Findings:**

This randomized clinical trial found that videoconference-based acceptance and commitment therapy (compared with a waiting list) was effective in reducing posttraumatic stress symptoms in parents of very ill children.

**Meaning:**

This study supports the use of acceptance and commitment therapy as an approach to reduce posttraumatic stress symptoms in parents of very ill children, following an acute or unexpected illness or diagnosis, and finds that a videoconferencing platform can be used effectively to access hard-to-reach populations.

## Teaser

This randomized clinical trial evaluates the efficacy of an acceptance and commitment therapy–based group intervention, delivered using videoconferencing, in reducing posttraumatic stress symptoms in parents of children with life-threatening illness.

## Introduction

Parents of children diagnosed with a life-threatening illness or injury are faced with significant psychosocial demands that may challenge their own psychological well-being. Although most parents adapt with time, a proportion suffer from psychiatric conditions, including posttraumatic stress disorder, as a direct result of their child’s illness.^1,2^ Others experience subthreshold, but clinically significant, psychiatric symptoms that can lead to longer-term mental health problems.^3,4^ Parental mental health problems may have implications for long-term psychological, behavioral, and emotional problems for the child,^5,6,7,8^ because high levels of distress can impair the parent’s capacity to respond to the demands of their child’s illness^9^ and can affect the home environment after discharge.^10^ A systematic review^9^ of family adjustment to childhood cancer described the complex effects of child illness on family life, including the lack of time for nonessential activities, the needs of the child who is ill taking priority over those of parents and siblings, and extended periods of family separation during treatment. Importantly, these clinically significant distress reactions in parents have been found across many illnesses, including children who have experienced trauma,^11,12^ those admitted to pediatric or neonatal intensive care units,^13,14,15^ and those newly diagnosed with type 1 diabetes.

Interventions targeting these early distress reactions in parents are critical, because these initial reactions have been found to be associated with long-term outcomes.^3^ This is consistent with the Pediatric Medical Traumatic Stress Model,^16,17^ which provides a useful framework in which to understand important associated factors and the development and trajectory of psychological disorders in parents associated with child medical trauma, as well as identifying the optimal time for treatment.

Despite the emotional vulnerability of this population, evidence-based, preventive mental health interventions are lacking. The few studies^18,19^ that have attempted to develop and test interventions have demonstrated limited success, with some evidence supporting problem-solving and family therapy approaches in parents of children with cancer.

Our study builds on promising pilot results^20,21^ to evaluate the effectiveness of Take a Breath, a brief group intervention for parents of children with a range of life-threatening illnesses and injuries. It is based on acceptance and commitment therapy (ACT)^22^ and comprises key elements of acceptance, mindfulness, values clarification, and goal setting. Given that intrusive thoughts, avoidance, and high levels of emotional arousal are among the most common distressing symptoms in parents of children with a serious illness or injury,^23,24^ the group-based ACT model was selected because it provides peer support and modeling of coping strategies and helps normalize the challenges faced by parents. The ACT-based intervention is delivered using videoconferencing to maximize accessibility for a highly stressed and geographically dispersed population.

This study aimed to determine the efficacy of the ACT-based program in reducing posttraumatic stress symptoms (PTSS) in parents. It was hypothesized that the program would result in a significant reduction in PTSS in the intervention group compared with a control group assigned to a waiting list for the intervention. The study also investigated whether the ACT-based program resulted in improvements in the psychological skills addressed in the intervention, such as mindfulness, values-based living, and psychological flexibility. Secondary outcomes explored whether there were improvements in other aspects of parental well-being, specifically, the parent’s experience of their child’s illness and family functioning. We hypothesized that the intervention group would show significantly better functioning within these areas compared with the waiting list group.

## Method

### Study Design

Ethics approval for this randomized clinical trial was granted by the Royal Children’s Hospital Human Research Ethics Committee. All procedures contributing to this work comply with the ethical standards of the relevant national and institutional committees on human experimentation and with the Declaration of Helsinki of 1975, as revised in 2008.^47^ Written informed consent was obtained from eligible parents. This study follows the Consolidated Standards of Reporting Trials (CONSORT) reporting guideline. The full protocol is available in Supplement 1 and has been published elsewhere.^25^

Eligible participants were randomized to either an intervention or a waiting list control group. Parents completed questionnaires at 3 time points: within 4 weeks of their child’s diagnosis or admission (T1, screening), 2 weeks before intervention commencement, or approximately 4 to 6 months after the diagnosis or admission (T2, preintervention), and immediately after intervention completion (T3, postintervention).

### Participants

Participants were parents of children with a recently diagnosed life-threatening illness or injury admitted to the oncology or cardiology departments or the pediatric intensive care unit (PICU) at the Royal Children’s Hospital, Melbourne, Australia. Admissions into these departments were chosen because of the sudden onset of the child’s illness and the significant threat posed to the child’s life and future functioning. Participants were recruited from consecutive first admissions to these departments from January 2014 to February 2016. Eligible participants were (1) the primary caregiver (aged ≥18 years) of a child aged 0 to 18 years; (2) parents of a child with a first-time presentation for a new cancer diagnosis, cardiac surgery within the first month of life, or admitted to the PICU for more than 48 hours; (3) parents reporting elevated levels of acute stress symptoms as measured by the Acute Stress Disorder Scale (ASDS)^26^; and (4) parents who were sufficiently fluent in English to complete questionnaires and participate in the program. Parents were excluded if they presented with a pre-existing psychiatric disorder, they had experienced another trauma (eg, death of family member) in the 2 months before the child’s diagnosis, they were no longer the primary caregiver at the time of the intervention, or their child was deemed eligible for palliative care (as determined by the hospital clinical care team) or had died.

### Procedure

The child’s medical team was consulted to determine study eligibility. Parents were approached on the hospital wards (or by telephone if the child had been discharged) by the research team to discuss participation and to screen for eligibility. Screening included completion of the ASDS^26^ and collection of demographic information at T1. At 4 months after admission, eligible parents were contacted by telephone and invited to take part in the trial. The intervention was not undertaken in the early stages of admission to allow for natural recovery of parents’ initial acute stress reactions. The timing of the ACT-based intervention is consistent with the Kazak Pediatric Medical Traumatic Stress Model.^4^ In some families, both parents were eligible; however, randomization occurred at the family level. Thus, parents from the same family were always in the same condition. Partners of eligible parents who did not meet eligibility criteria were also invited to participate because they were seen as a key source of support for enacting changes^27^ and may have also benefited from the program. Data from parents who were invited but not eligible were not included in analyses.

Parents were asked to consent to trial participation and were enrolled at T2 before randomization. The Murdoch Children’s Research Institute’s Clinical Epidemiology and Biostatistics Unit, independently of the research team, generated the randomization list with a computerized randomization plan generator using the method of randomly permuted blocks. Three randomly allocated lists were created, 1 for each hospital department (cardiology, oncology, and PICU). A researcher independent of participant recruitment and intervention delivery managed the lists and allocated parents to intervention or the waiting list using sequentially numbered, sealed envelopes. This process ensured that parents and the research team were blind to participant allocation at assessment. Once the intervention was completed, both the intervention and waiting list groups completed their surveys at T3.

### Intervention or Treatment

The program is a 6-session, parent-mediated, psychological intervention based on ACT.^25^ It consists of five 90-minute consecutive weekly sessions, with a sixth and final session held 3 weeks after the fifth session. Parents participated from their homes using the Google Hangouts videoconferencing application (Alphabet) on a study-provided iPad (16 GB with Retina Display Wi-Fi +3G; Apple).

Each ACT session was delivered by 2 trained mental health clinicians. Session content and structure are reported in eTable1 in Supplement 2. Intervention materials (a set of values cards, a session booklet, and guided mindfulness CD and MP3 file) were sent to parents to enhance online participation. Each ACT group consisted of a maximum of 8 parents and partners, and each participant was able to interact via video with the facilitators and other participants. Each group included parents across different stratified illness groups. Waiting list participants received standard medical treatment and allied health support that was routinely provided within their treating teams throughout their treatment.

### Facilitator Training and Program Fidelity

A total of 4 facilitators received training in ACT and in the delivery of the intervention from a senior ACT clinician, who is a specialist in trauma-focused psychotherapy. Each session was audio recorded and reviewed fortnightly during team supervision sessions. Fidelity of program delivery was recorded and assessed using session monitoring checklists completed by both facilitators after each session. An audio recording of 1 session from each group was randomly selected and reviewed for fidelity by an independent clinician, using a fidelity monitoring checklist.

### Screening Measures

The ASDS and a demographic information questionnaire were completed at T1 only. All other measures were completed at T2 and T3 by parents in both groups. Each measure has been evaluated as a valid and reliable measure in these clinical populations.

The ASDS^26^ measures acute stress reactions within 4 weeks after a traumatic event and is highly predictive of the development of longer-term PTSS and mental health problems.^3,28^ The ASDS measures symptoms of dissociation, re-experiencing, avoidance, and arousal and can be scored using a total score cutoff (a score ≥56 was recommended for identifying individuals at risk of later developing posttraumatic stress disorder)^26^ or in terms of symptom clusters. A score of 9 or higher on the dissociative cluster, as well as a score of 28 or higher on the combined re-experiencing, avoidance, and arousal cluster scores, indicates the presence of clinically significant levels of acute stress disorder symptoms^26^. Both scoring methods were used in this study to determine eligibility, with parents required to be eligible on at least 1 scoring method to be invited to participate. Internal consistency (Cronbach α) was 0.80 for the ASDS total score, 0.71 for dissociation, 0.32 for re-experiencing, 0.75 for avoidance, and 0.64 for arousal.

### Primary and Secondary Outcomes

The primary outcome was PTSS in parents as measured by the Posttraumatic Stress Disorder Checklist–Version 5 (PCL-5).^29^ It contains 20 items that assessed 20 criteria for posttraumatic stress disorder in the *Diagnostic and Statistical Manual of Mental Disorders* (Fifth Edition).^30^ Parents were asked to complete the measure in relation to their child’s diagnosis. The total score (range, 0-80) was used, with higher scores indicating greater PTSS. The PCL-5 has been found to be a valid and reliable measure across many populations.^29,30,31^ Internal consistency for the total score in the current study was α = .93. Secondary outcomes measured included depression, anxiety, and stress (Depression, Anxiety, and Stress Scale–21)^32,33^; the parents’ adjustment and experience of the illness (Parent Experience of Child Illness Scale [PECI])^34^; and the family’s ability to manage caring for a child with a chronic condition (condition management ability subscale of the Family Management Measure).^35^

### Functional Outcomes: Psychological Skills Addressed by the Intervention

Changes in the psychological skills taught within the intervention were also evaluated. These included acceptance (Acceptance and Action Questionnaire–II),^36,37^ various aspects of day-to-day mindfulness (Five Facet Mindfulness Questionnaire–Short Form),^38,39^ values-based living and the degree to which people live by their values (Valuing Questionnaire),^40^ and the level of psychological flexibility in parents (Parental Psychological Flexibility Questionnaire).^41,42^

### Demographic and Medical Information

Information was collected regarding parent age, sex, marital and employment status, level of education, and country of birth, as well as their child’s age and sex. Medical diagnosis, date of diagnosis, and treating department were extracted from hospital medical records.

### Statistical Analysis

To detect a difference of 7.5 (SD, 13.94) in the primary outcome measure (PCL-5), between the 2 treatment groups, with a significance level of 2-sided *P* < .05 and power of 0.80, 72 participants were required per group. Allowing for an estimated 35% lost to follow-up, it was projected that a total of 210 participants was needed to be randomized across the 2 groups.

Demographic, illness group (oncology, PICU, or cardiac), and ASDS screener characteristics were compared between groups. For parent-level data (for couples that were both eligible), categorical variables were compared using generalized estimating equation models to account for within-couple correlation (with the exception of parent sex and illness group). A generalized estimating equation with a Gaussian distribution was used to compare group means of the ASDS screener. For child-level characteristics, child ages at diagnosis and at each subsequent time point were compared using negative binomial regression models because of positive skew. Time between measurements using linear regression models and illness group distributions were compared using a χ^2^ test.

Generalized estimating equation models also compared mean outcome measures between groups at T3, adjusting for scores at T2 (included as a model covariate). Again, models accounted for within-couple correlations. Effect size Cohen *d* values were calculated between groups for T3 scores (adjusted for T2 scores) and were interpreted as small (<0.2), medium (0.2 to <0.5), large (0.5 to 0.8), and very large (>0.8).^43^ These analyses were all stratified by illness group.

All analyses were performed using Stata statistical software version 15.1 (StataCorp). Two-sided statistical significance was set at *P* < .05. To not unduly penalize the data, multiple comparison adjustment was not applied. Data analysis was performed from July to September 2018.

## Results

The recruitment flow of participants can be seen in the Figure. In total, 1232 parents were assessed for eligibility, and 313 were randomized (161 to the waiting list control group and 152 to the intervention group). A total of 114 of 313 parents (36.4%) who initially consented and were randomized completed the questionnaire at T2 and were enrolled in an intervention, for an attrition rate of 64%. Of those allocated, 44 parents in the waiting list group and 37 in the intervention group completed the postintervention questionnaire and were analyzed (81 participants total; mean [SD] age, 37.17 [6.43] years). Sixty-five participants (80.2%) were women, 48 (59.3%) were married, and 40 (49.4%) lived in rural or regional areas, or in a different state. In addition, 24 participants (29.6%) were in the cardiology illness group, 32 participants (39.5%) were in the oncology group, and 25 participants (30.9%) were in the PICU group.

**Figure.**
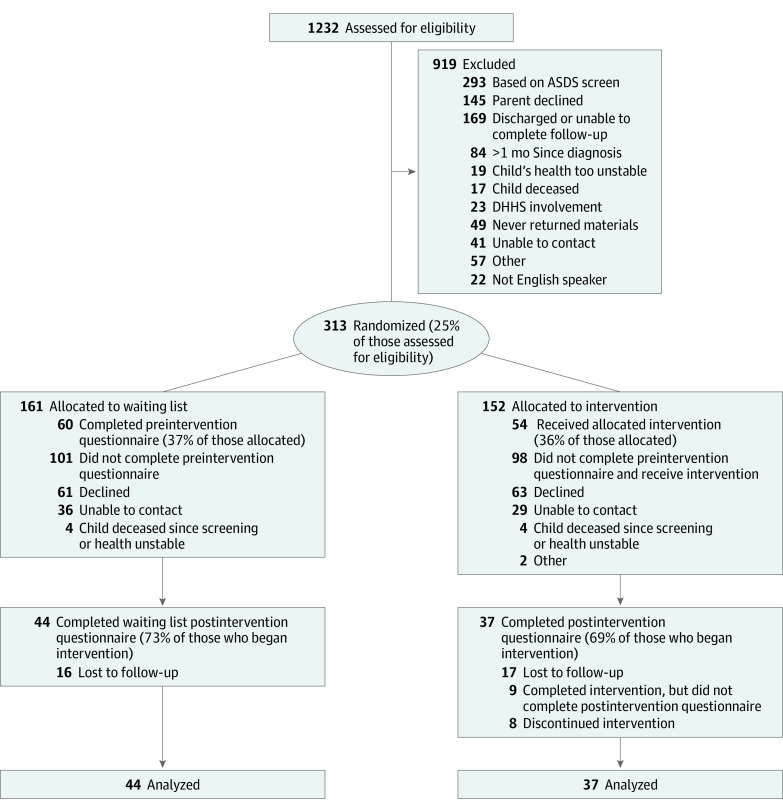
The Recruitment Flow of Participants ASDS indicates Acute Stress Disorder Scale; DHHS, Department of Health and Human Services.

Reasons for nonenrollment included parents being unable to be contacted (65 parents [20.8%]), declining participation for various reasons, including time constraints (124 parents [39.6%]), and child death or unstable health since screening (8 parents [2.6%]). Results from between-group analyses are presented in Table 1, with no differences found between groups. Most parents were women (34 parents [77.3%] in the waiting list group vs 31 parents [83.8%] in the intervention group) and married (26 parents [59.1%] in the waiting list group vs 22 parents [59.9%] in the intervention group), and similar numbers were employed full time (11 parents [25.0%] in the waiting list group vs 7 parents [18.9%] in the intervention group). The mean (SD) scores on the ASDS at screening were 57.4 (10.4) for the waiting list group and 56.8 (11.4) for the intervention group.

**Table 1.** Demographic Comparison of Study Groups

Characteristic	Participants, No. (%)		*P* value^a^
	Waiting list control group (n = 44)	Intervention group (n = 37)	
Parents			
Age, mean (SD), y	36.45 (6.74)	38.04 (6.02)	.27
Sex			
Male	10 (22.7)	6 (16.2)	.46
Female	34 (77.3)	31 (83.8)	
Marital status^b^			
Single	2 (4.5)	1 (2.7)	.87
Married	26 (59.1)	22 (59.5)	
Living with partner	6 (13.6)	5 (13.5)	
Missing	10 (22.7)	9 (24.3)	
Employment status^b^			
Full time	11 (25.0)	7 (18.9)	.18
Part time	10 (22.7)	3 (8.1)	
Casual	2 (4.5)	6 (16.2)	
Not employed	7 (15.9)	11 (29.7)	
Parental leave	3 (6.8)	1 (2.7)	
Missing	11 (25.0)	9 (24.3)	
Highest education^b^			
Postgraduate degree	12 (27.3)	5 (13.5)	.80
Graduate diploma or graduate school	2 (4.5)	5 (13.5)	
Bachelor’s degree	9 (20.5)	7 (18.9)	
Advanced diploma or diploma	7 (15.9)	4 (10.8)	
Certificate	5 (11.4)	9 (24.3)	
Year 12	6 (13.6)	4 (10.8)	
Year 11 or below	3 (6.8)	3 (8.1)	
Aboriginal or Torres Strait Islander	1 (2.3)	0	>.99
Missing	2 (4.5)	1 (2.7)	
Language other than English at home^b^	6 (13.6)	3 (8.1)	.78
Born in Australia^b^	37 (84.1)	27 (73.0)	.77
Missing	0	1 (2.7)	
Acute Stress Disorder Scale score at screening, mean (SD)^b^	57.4 (10.4)	56.8 (11.4)	.86
Illness group			
Cardiology	17 (38.6)	7 (18.9)	.15
Oncology	15 (34.1)	17 (45.9)	
Pediatric intensive care unit	12 (27.3)	13 (35.1)	
Children	40	34	
Age before intervention, median (interquartile range), y^c^	1.6 (0.9-5.2)	2.9 (1.2-10.3)	.23
Age after intervention, median (interquartile range), y^c^	1.8 (1.0-5.3)	3.2 (1.4-10.5)	.22
Interval between before and after intervention, mean (SD), mos	11.4 (3.5)	11.2 (3.6)	.89
Age at diagnosis, median (interquartile range), y^c^	0.7 (0-4.4)	2.1 (1.5-9.9)	.32
Illness group			
Cardiology	16 (40.0)	6 (17.6)	.11
Oncology	13 (32.5)	15 (44.1)	
Pediatric intensive care unit	11 (27.5)	13 (38.2)	

^a^ Missing values were excluded from *P* value calculations.

^b^ Generalized estimating equation model, clustered by family.

^c^ Negative binomial distribution.

Preliminary analyses also explored the differences between participants who consented and participated, and those who consented but did not participate across these same sociodemographic factors (eTable 2 in Supplement 2). There were differences between the groups regarding sex, with a greater proportion of men dropping out (48 parents [34.0%] vs 16 parents [19.8%]), as well as the primary language spoken at home, with families whose primary language was not English more likely to drop out (34 parents [24.1%] vs 9 parents [11.1%]). No other significant differences were identified. Approximately half of the sample (41 parents [50.6%]) lived in metropolitan Melbourne, and half (40 parents [49.4%]) lived in rural or regional areas or in other Australian states.

Fidelity of program delivery was rated at 98% across all sessions. There was a total of 26 groups, with group sizes ranging from 3 to 8 parents. Table 2 shows the mean scores for all outcome measures at both the T2 and T3 time points, across both groups. Analyses compared T3 group means, adjusting for T2 scores as well as illness group, with the latter being nonsignificant in all models. Significant differences were found between the intervention and waiting list groups at T2 across many outcome measures, with the ACT-based intervention group commencing the trial with fewer symptoms and higher functioning in many areas compared with the waiting list group, including PCL-5 total (mean score, 23.3 [95% CI, 18.6-28.1] vs 31.7 [95% CI, 27.0-36.4]; *P* = .03), PECI unresolved sorrow and anger (mean score, 1.3 [95% CI, 1.1-1.6] vs 1.8 [95% CI, 1.6-2.1]; *P* = .004), PECI uncertainty (mean score, 1.5 [95% CI, 1.2-1.8] vs 2.0 [95% CI, 1.8-2.3]; *P* < .001), and PECI negative appraisal (mean score, 1.6 [95% CI, 1.4-1.8 vs 2.1 [95% CI, 1.8-2.3; *P* = .03). Preintervention functioning at T2 was therefore controlled for in subsequent analyses as a model covariate. Concerning the investigation of the differences between the intervention and waiting list groups from T2 to T3, significant differences were found on the primary outcome measure (PCL-5), after accounting for preinjury functioning at T2, with greater improvements in the PTSS after the intervention. For the intervention group, mean PTSS scores decreased from 23.3 (95% CI, 18.6-28.1) at T2 to 17.8 (95% CI, 13.8-21.8) at T3. For the waiting list group, mean PTSS scores decreased from 31.7 (95% CI, 27.0-36.4) at T2 to 26.2 (95% CI, 21.8-30.7) at T3 waiting list (Cohen *d* = 1.10; 95% CI, 0.61-1.59; *P* = .03), for a mean reduction of 5.5 points for both groups. Significantly greater improvements were also found at T3 for the experience and impact of the illness on parents (ie, PECI), with greater improvements found in the emotional resources (Cohen *d* = 0.95; 95% CI, 0.48-1.42; *P* = .002), uncertainty (Cohen *d* = 1.34; 95% CI, 0.84-1.83; *P* < .001), and the overall negative appraisal subscales (Cohen *d* = 0.98; 95% CI, 0.51-1.44; *P* = .03). No additional group differences were identified in the other mental health (ie, Depression, Anxiety, and Stress Scale–21), family functioning (ie, Condition Management Ability Subscale of the Family Management Measure), and other parent experience measures (ie, PECI unresolved anger and guilt and worry subscales).

**Table 2.** Scores for Main Outcomes Between Groups Before and After the Intervention

Outcome measures and time points	Waiting list control group		Intervention group		Cohen *d* (95% CI)^a^	*P* value^b^
	Parents, No.	Score, mean (95% CI)	Parents, No.	Score, mean (95% CI)		
Posttraumatic Stress Disorder Checklist–Version 5 total						
T2	41	31.7 (27.0-36.4)	34	23.3 (18.6-28.1)	1.10 (0.61-1.59)	.03
T3		26.2 (21.8-30.7)		17.8 (13.8-21.8)		
Depression, Anxiety, and Stress Scale						
Depression						
T2	44	11.3 (8.9-13.7)	36	8.3 (5.8-10.8)	0.51 (0.06-0.96)	.41
T3		10.6 (8.3-12.8)		7.7 (4.8-10.5)		
Anxiety						
T2	44	9.6 (7.2-12.0)	36	6.4 (4.2-8.7)	0.60 (0.15-1.05)	.69
T3		8.4 (5.7-11.0)		6.0 (4.1-7.9)		
Stress						
T2	44	18.0 (15.1-21.0)	36	15.9 (12.7-19.1)	0.44 (0-0.89)	.16
T3		16.6 (13.7-19.4)		13.2 (9.9-16.4)		
Parent Experience of Illness Scale						
Guilt and worry						
T2	43	2.3 (2.0-2.6)	36	1.9 (1.7-2.2)	0.74 (0.28-1.20)	.09
T3		2.1 (1.9-2.4)		1.7 (1.4-1.9)		
Emotional resources						
T2	44	2.2 (2.0-2.4)	35	2.5 (2.3-2.8)	0.95 (0.48-1.42)	.002
T3		2.3 (2.1-2.5)		2.8 (2.6-3.0)		
Unresolved sorrow and anger						
T2	44	1.8 (1.6-2.1)	36	1.3 (1.1-1.6)	0.82 (0.36-1.27)	.19
T3		1.8 (1.5-2.1)		1.2 (1.0-1.4)		
Uncertainty						
T2	43	2.0 (1.8-2.3)	35	1.5 (1.2-1.8)	1.34 (0.84-1.83)	<.001
T3		2.0 (1.7-2.3)		1.2 (1.0-1.4)		
Negative appraisal						
T2	44	2.1 (1.8-2.3)	36	1.6 (1.4-1.8)	0.98 (0.51-1.44)	.03
T3		1.9 (1.7-2.2)		1.4 (1.2-1.6)		
Family Management Measure, condition management ability						
T2	44	41.6 (39.7-43.5)	34	43.2 (41.3-45.2)	0.60 (0.14-1.05)	.13
T3		41.9 (40.1-43.7)		44.2 (42.7-45.7)		

Abbreviations: T2, 2 weeks before intervention commencement, or approximately 4 to 6 months after the diagnosis or admission; T3, immediately after intervention completion.

^a^ Cohen *d* values are for postintervention scores between groups, adjusted for preintervention scores.

^b^ Mean comparison between groups at follow-up using generalized estimating equation models (clustering on dyad), adjusting for illness group and preintervention score.

Functional outcomes are displayed in Table 3, which report differences between the intervention and waiting list groups in the psychological skills taught in the intervention, from T2 to T3. The intervention group displayed improvements that were of a significantly larger magnitude in experiential avoidance (Acceptance and Action Questionnaire–II; specifically that parents were less avoidant and more flexible) (Cohen *d* = 0.80; 95% CI, 0.33-1.27; *P* = .02), as well as greater improvements in mindfulness, specifically in nonjudging of inner experience (Five Facet Mindfulness Questionnaire–Short Form) (Cohen *d* = 1.51; 95% CI, 0.99-2.01; *P* = .006), living according to values (Valuing Questionnaire) (Cohen *d* = 1.14; 95% CI, 0.65-1.62; *P* = .006), and committed action (Parental Psychological Flexibility Questionnaire) (Cohen *d* = 0.86; 95% CI, 0.39-1.34; *P* = .03). There were no other significant improvements in the other functional outcomes from T2 to T3.

**Table 3.** Functional Outcomes Scores Between Groups, Before and After Intervention

Outcomes measures and time points	Waiting list control group		Intervention group		Cohen *d* (95% CI)^a^	*P* value^b^
	Parents, No.	Score, mean (95% CI)	Parents, No.	Score, mean (95% CI)		
Acceptance and Action Questionnaire–I total						
T2	44	24.9 (22.4-27.5)	32	22.9 (20.5-25.3)	0.80 (0.33-1.27)	.02
T3		25.3 (22.3-28.2)		20.1 (17.2-22.9)		
Five Facet Mindfulness Questionnaire–Short Form						
Observing (noticing)						
T2	44	12.3 (11.3-13.4)	34	12.2 (10.8-13.5)	0.19 (0.26-0.64)	.52
T3		12.7 (11.6-13.8)		13.1 (12.1-14.0)		
Describing (labeling)						
T2	44	16.1 (15.2-17.1)	34	17.4 (16.2-18.7)	0.19 (0.26-0.64)	.33
T3		16.6 (15.4-17.7)		17.7 (16.2-19.2)		
Nonreactivity to inner experience						
T2	44	14.3 (13.2-15.5)	34	14.6 (13.6-15.7)	0.38 (0.08-0.83)	.17
T3		15.1 (14.0-16.3)		16.0 (15.0-17.1)		
Acting with awareness						
T2	44	14.8 (13.7-15.8)	34	14.9 (13.7-16.1)	0.66 (0.20-1.12)	.09
T3		14.5 (13.3-15.7)		15.8 (15.0-16.7)		
Nonjudging of inner experience						
T2	44	14.2 (13.3-15.1)	34	15.6 (14.5-16.7)	1.51 (0.99-2.01)	.006
T3		14.2 (13.3-15.1)		16.6 (15.6-17.6)		
Valuing Questionnaire						
T2	42	3.6 (3.4-3.9)	34	3.9 (3.6-4.1)	1.14 (0.65-1.62)	.006
T3		3.6 (3.4-3.9)		4.2 (3.9-4.4)		
Parental Psychological Flexibility Questionnaire						
Acceptance						
T2	43	4.5 (4.3-4.7)	33	4.5 (4.3-4.8)	0.18 (0.27-0.64)	.31
T3		4.6 (4.3-4.8)		4.5 (4.2-4.8)		
Cognitive defusion						
T2	42	5.3 (5.0-5.6)	33	5.5 (5.2-5.9)	0.65 (0.18-1.11)	.13
T3		5.1 (4.8-5.5)		5.5 (5.2-5.9)		
Committed action						
T2	43	5.1 (4.8-5.4)	33	5.2 (4.8-5.5)	0.86 (0.39-1.34)	.03
T3		5.1 (4.9-5.4)		5.4 (5.2-5.7)		

Abbreviations: T2, 2 weeks before intervention commencement, or approximately 4 to 6 months after the diagnosis or admission; T3, immediately after intervention completion.

^a^ Cohen *d* values are for postintervention scores between groups, adjusted for preintervention scores.

^b^ Mean comparison between groups at follow-up using generalized estimating equation models (clustering on dyad), adjusting for illness group and preintervention score.

## Discussion

The Take a Breath program is an ACT-based group intervention designed to prevent and reduce parents’ PTSS after the diagnosis of a serious illness in their child. Delivery via online videoconferencing sought to increase accessibility. To our knowledge, this randomized clinical trial provides the first evidence internationally to support the use of this type of approach for parents of children with diverse illnesses. Before the intervention, parents randomly allocated to the intervention condition were significantly less distressed across a range of measures than parents allocated to the waiting list control group. On our primary outcome measure, parents in both the intervention and control conditions had a mean reduction on their PTSS score of 5.5 points between T2 and T3. This represented a significantly larger relative reduction for intervention parents when their lower initial distress was taken into account. On the secondary outcome measures, parents in the intervention group showed significantly greater improvements than parents in the control group on 3 aspects of their subjective experience of the illness: improved emotional resources to manage the illness, reduced perceptions of uncertainty, and reduced negative appraisal associated with their child’s illness. Inspection of the measures of psychological skills addressed by the intervention suggests that these improvements may be associated with greater acceptance, mindfulness (specifically, nonjudging inner experiences), values-based living, and committed action in parenting flexibility. Together, these results suggest that participation in the intervention improved parents’ capacity to better manage the demands of caring for a sick child and potentially ameliorated the negative impact of parental PTSS on the child.^5^

The application of an ACT-based intervention is highly novel in this population, with past studies using traditional cognitive behavioral therapy^18,19,43,44^ or problem-solving therapy.^18,24^ Although we found significantly greater improvements in PTSS for parents receiving the 6-session intervention, there was no difference between groups for our secondary mental health measures of anxiety, depression, and stress. It is possible that a longer intervention is required to successfully address these additional mental health concerns; however, the program was not designed to address these symptoms. Past intervention studies in this field have all focused on the parents of children with cancer.^14,15,16,21,41^ The ACT-based intervention was beneficial regardless of the child’s illness or injury, providing support for intervention approaches that target parents across the wider hospital system. The findings also support the use of videoconferencing as a promising approach to enhance accessibility, which is a key barrier to the provision of evidence-based treatments for this population.^45^ Approximately half of our participants lived in rural or regional Victoria, Australia, or in another state. Use of the videoconferencing platform provided access to these parents who would not have been able to attend a traditional face-to-face service. Engaging parents within a tertiary hospital setting has been recognized as particularly challenging.^20,23,44,46^ Our approach also demonstrates the feasibility of using videoconferencing for a group program to provide opportunity for peer support and the normalization of experiences.

### Limitations

Implementation of this trial presented many challenges, and our study has some limitations. Recruiting parents when their child was seriously ill was difficult, and it took 2 years to recruit the sample despite working within a large tertiary hospital. As a result, our modest sample size meant that we were only able to detect large differences between the intervention and control groups. In addition, attrition rate of parents initially screened and enrolled but then lost to follow-up before commencement of the program was 64%. Primary reasons for attrition at this stage were time constraints or feeling that intervention was not needed. Fathers and parents whose primary language was not English were overrepresented in the study dropouts. This finding is not uncommon in intervention studies and further highlights the need to ensure that recruitment and intervention materials reflect diversity and are engaging to all.^23,46^ Other limitations that may have biased the findings include that the study only used self-reported measures and that, at the time of postintervention assessment at T3, the participants were no longer blinded as to group allocation. Additionally, multiple comparison adjustments were not applied to secondary and functional outcomes, potentially inflating the type I error rate. Furthermore, significant baseline differences in mental health symptoms between the intervention and waiting list groups indicate that randomization did not successfully allocate equivalent groups to treatment groups. It is unclear why this occurred; hence, replication of differential postintervention improvements using a sample with equivalent groups at baseline is required to substantiate the important findings reported here.

## Conclusions

The findings of this randomized clinical trial support the use of an ACT approach to reduce PTSS in parents of children who are seriously ill, across various illness groups, indicating that Take a Breath has potential for wide use within the pediatric hospital system. The findings also suggest that a videoconferencing format can be used in a clinically effective way to access geographically dispersed populations and engage their participation in a group program.

## Notes

Supplement 1. **Trial Protocol**

Supplement 2. **eTable 1.** Take a Breath Session Content and Structure **eTable 2.** Comparison Between the Analytic Sample and Those That Consented but Were Lost/Dropped Out

Supplement 3. **Data Sharing Statement**
