# Supplementary material for: Effect of a Videoconference-Based Online Group Intervention for Traumatic Stress in Parents of Children With Life-threatening Illness: A Randomized Clinical Trial
Source: JAMA Netw Open. 2020 Jul 31;3(7):e208507. doi: 10.1001/jamanetworkopen.2020.8507 (PMC7395233; doi:10.1001/jamanetworkopen.2020.8507)
Supplement: Supplement 1. — Trial Protocol [file jamanetwopen-3-e208507-s001.pdf]

A Randomised Controlled Trial of the "Take a Breath" Parent Program: Evaluation of A Program to Reduce Distress in Parents of Children with Serious Childhood Illnesses/injuries

2. INTRODUCTION AND BACKGROUND

2.1 Background Information

It has been well established that serious child illness or injury's (SCII) which are life threatening and have long term consequences for child wellbeing, have significant impacts on child health and development, including social, educational, physical and emotional domains. Importantly, these increased problems occur not just for the sick child (Caplan et al., 2005; Hudson et al., 2003; Zebrack et al., 2002) but also for parents, and for mothers in particular (Pai et al., 2007; Pai et al., 2008). Parents of a child with an SCII must, for example, contend with the possibility of their child's death along with the serious impact on their child's future (Hall et al., 2006). These experiences can overwhelm even the most resilient parents (Hall et al., 2006).

Research findings from studies exploring the impact of SCII have indicated that most families of a child with SCII are able to cope and adjust well over time despite initial and/or recurrent periods of extreme distress (A. Kazak et al., 2007; Landolt, Vollrath, Ribi, Gnehm, & Sennhauser, 2003; Tyack & Ziviani, 2003). However, it has also been documented that for some families, family functioning will be adversely affected by elevated or escalating psychological distress in parents (Chesire, Barlow, & Powell, 2010; Mu, 2005; Shaw et al., 2006; Williams et al., 2003). Similar to other traumatic experiences, the experience of having a child diagnosed with a SCII can lead to parental depression, acute stress disorders and/or post traumatic stress responses (Best, Streisand, Catania, & Kazak, 2001; Chesire et al., 2010; Cleveland, 2008; A. Kazak & Barakat, 1997). This link between parental mental health and SCII has been seen across a range of diagnoses including: cancer, acquired brain injury and admission to Intensive Care Units (Sawyer, Streiner, Antoniou, Toogood, & Rice, 1998).

Research suggests that the early months of treatment provide an important window to intervene to reduce short and longer-term post traumatic symptoms in parents (A. Kazak et al., 2007). Some attention has been given to the investigation of approaches for supporting parents of children with SCII and in particular childhood cancer. Many of these approaches have targeted parents with existing symptoms of trauma and distress. Results of studies have been mixed. While some studies have demonstrated significant impacts on parental coping and wellbeing (A Kazak et al., 1999; O. Sahler et al., 2002), other studies have reported interventions to be less effective than required to support wide dissemination. Two studies aimed at reducing parental symptoms of traumatic stress in parents of children with cancer have demonstrated some success. The first, the Surviving Cancer Competently Intervention Program (SCCIP: (A Kazak et al., 1999) is a one day group cognitive behavioural and family systems intervention whilst the second is a problem solving skills training program, (PSST: (O. Sahler et al., 2002) delivered on an individual basis to parents over eight

sessions. Findings from families participating in either the SCCIP or PSST interventions revealed both programs were effective in reducing maternal emotional distress and anxiety associated with the diagnoses of life-threatening illnesses. However, both these interventions report some problems, with the SCCIP reporting difficulties with engagement of parents and the outcomes from the PSST program diminishing at three month follow-up assessment. Another study, evaluated the effectiveness of a parent component of the four session Children's Epilepsy Program (Lewis, Hatton, Salas, Leake, & Chiofalo, 1991). The parent component was designed to help parents acknowledge their fears, grief, and anger surrounding their child's seizure disorder, as well as to have a better understanding of the disorder (Lewis et al., 1991). The results from the pilot study revealed gains in parental knowledge and reductions in anxiety, but did not result in changes to parental vigilance and the restrictions they placed upon their child's self-care activities or how they controlled their seizures, a major aim of the intervention (Lewis et al., 1991). The sparse amount of intervention research to date and the mixed results of the few published studies highlight the need for further investigation and development of effective interventions that prevent traumatic stress symptoms in parents from reaching clinical levels.

More recently psychosocial interventions are moving into online platforms to enhance the feasibility of research studies, and to provide rural and regional families access to potentially useful programs. Large tertiary paediatric hospitals like Melbourne's RCH receive admissions from across the state as well as from interstate and overseas. Our longitudinal study of 194 parents of children with SCIIs found 46% lived in regional, rural or interstate areas. To ensure equity of access the use of an online videoconferencing is a viable option. The efficacy of telepsychiatry and other technology-assisted psychological interventions services has been studied for over 15 years. While the number of quality RCTs remains small, the evidence consistently supports these approaches as having effect sizes equal to and sometimes stronger than traditional face-to-face intervention. Meta-analyses of technology-assisted interventions for the treatment of adult anxiety, depression, or trauma span interventions delivered individually or in groups, using on-line text-based intervention, tele- and videoconferencing for groups or individuals, and self-help programs with or without therapist assistance (Barak, Hen, Boniel-Nissim, & Shapira, 2008). There is also growing evidence that elements of the therapeutic alliance (which arguably account for a substantial proportion of outcomes irrespective of the intervention components),(Frueh et al., 2007) remain similar regardless of mode of delivery (Cook & Doyle, 2002; Frueh et al., 2007). For example, Frueh et al (2007) evaluated a manualised intervention for PTSD (N=38) and found no differences by modality (group teleconferencing) in terms of therapist competence, adherence, providing feedback, developing rapport, managing difficulties and conveying empathy.

2.3 Rationale for Current Study

The newly developed Take a Breath (TAB) parent program, which targets parents at risk for developing serious psychosocial difficulties, offers an important opportunity to provide tailored support to families with the potential to prevent ongoing difficulties and to assist families to adapt well to the new and often overwhelming experience of having a seriously ill child. The program incorporates key strategies that have demonstrated promise in previous intervention trials for parents of children with an SCII whilst adding additional therapeutic elements that have demonstrated good effect in other areas of psychological trauma and distress. It is anticipated that the combination of these therapeutic elements will lead to

prevention of ongoing and severe levels of traumatic stress in vulnerable parents of children with a range of serious childhood illnesses.

3. STUDY OBJECTIVES

3.1 Primary Objective

Does the "Take A Breath program lead to greater improvements in psychosocial distress than treatment as usual for parents of children with SCIIs who report risk factors associated with the development of post traumatic stress symptoms in the first weeks following their child's diagnosis.

4. STUDY DESIGN

4.1 Type of Study

The design is a pre-post- control group design with random allocation to one of two treatment arms:

- TAB (Take a Breath) parent intervention
- Wait List control (W-L).

This design will allow a comparison of the TAB and wait-list groups at pre and post intervention, in order to evaluate if any improvements over time are due to the TAB intervention and not due to natural recovery or other factors. Following completion of the post questionnaire, the W-L group will be offered the TAB intervention if they are interested, to acknowledge their time commitment and participation in the project. If they do complete the group, they will also be asked to complete a post-intervention questionnaire.

Up to 30 parent groups will be delivered per arm across a two and a half year period commencing in November 2014. Parents assigned to the TAB arm will be offered a place in the next available parent group. Parents assigned to the W-L arm will be offered participation in the TAB program immediately after completion of the post intervention questionnaire.

4.3 Number of Subjects

Screening of Parents: Identifying Target Participants

Up to 788 parents will participate in the Screening process in order to reach the targeted group. It is anticipated that this number of parents will be required to reach the 263 eligible and consenting parents that are required for adequate power for the parent intervention analysis. This figure was arrived at by allowing for a seventy percent uptake at screening, a 5% drop due to child death/grave illness and 50% excluded as reporting no distress.

It is considered that this figure of 788 is feasible based on the number of past admissions from each of the three participating departments in a 12 month period. For example, during 2008-2009 there were approximately 200 new cancer, 1600 Cardiac, and there were approximately 1000 admissions to the PICU. Given these figures and the research experience of the research team, it was estimated that approximately 70% of families admitted into the hospital in each of the illness/injury groups would be likely to participate resulting in

sufficient numbers of families willing to complete the screening questionnaire. See figure 3 below outlying recruitment, assessment and participant flow.

Parent Intervention Participants

One hundred and eighty four families will be allocated to each of the two treatment arms. A potential total of 263 eligible parents are required, as we anticipate that 50% of eligible families will either be uncontactable, refuse to participate, or their child's illness will exclude them from participating at this later stage. See Figure 3 for participant flow details.

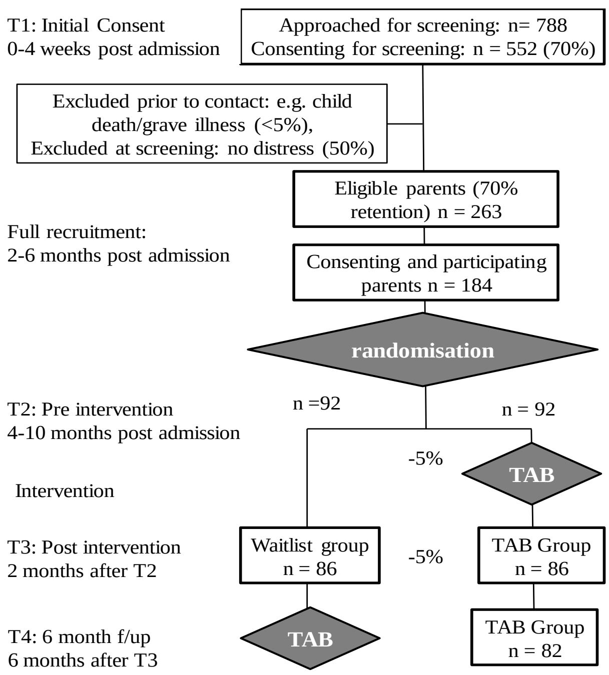

Figure 1: Recruitment, assessment & participant flow

Figure 3: Recruitment, assessment and participant flow

5. STUDY TREATMENTS

5.1 Treatment Arms

5.1.1 Description

Program Structure

The parent intervention is a brief psychosocial parent mediated group program. It will consist of five sessions of 90 minutes duration each plus one additional sixth session (also 90 minutes) one month following the fifth session (totalling nine hours of contact time). The intervention will be delivered via an online videoconferencing platform called Google Hangouts https://hangouts.google.com/supported by the Royal Children's Hospital. This

program allows participants and practitioner/s to see and hear each other, including the option of presenting teaching materials on the screen. Participants are also able to send group emails and private emails to the practitioner if they chose to do so. Each participant will be sent an I-pad (set up for immediate use) for the duration of the program, programmed with Google hangouts, and specific instructions on how to login to the session. The I-pad will have internet access, with ample download capacity with Telstra to prevent disruption throughout this period. Internet access will be restricted to the Google hangouts application, limiting extraneous use of the internet. This process will ensure all participants have access to the technology and will minimise technical issues.

Voice recordings will be taken in each group, and used to assess program fidelity. To maintain privacy, participants will be requested to enter their first name only, which will be visible underneath their webcam image. An overview of the program's structure and content is provided in Appendix 11: TAB Program Outline.

Program Approach

The parent program will be based on several key theoretical approaches that have been shown to be effective in supporting parents and/or in addressing a range of psychosocial difficulties.

First, the program will adopt strategies from a Cognitive Behavioural Psychology (CBT) approach. In particular, Social Learning Theory (also known as Behavioural Family Intervention or Parent Management Training), is an approach that has been applied extensively and effectively in the parenting field. This theory will inform both the learning processes and the program content. Social Learning Theory approaches utilise behaviour management training, modelling, and generalisation and maintenance strategies, such as in session and between session practice, to assist parents to achieve changes in their children's and their own behaviour. Positive outcomes include reductions in child internalising and externalising behaviour problems, increases in parental confidence and effectiveness, improvements in the psychosocial health of parents (including anxiety, depression and self esteem) and development of skills for improving parenting practices and improving the parent-child relationship quality (Johnson, Franklin, Hall, & Prieto, 2000; Sanders, Markie-Dadds, & Turner, 2003; Webster-Stratton & Hammond, 1997). In relation to SCIIs, interventions that have incorporated CBT strategies such as problem solving have been demonstrated to be the most effective to date (A. Kazak et al., 1998; O. J. Z. Sahler et al., 2002). Additionally in the area of SCIIs, such as acquired brain injury, parenting programs targeting difficult child behaviours are showing some promise (Feeney & Ylviksaker, 1995; Slifer et al., 1996).

In addition, the intervention will also incorporate strategies from a contextual-behavioural psychology approach. This approach also has its origins in the field of Cognitive Behavioural Psychology, is relatively new and has increasing levels of evidence for its effectiveness in addressing a range of psychological difficulties, including, depression and anxiety in adults and children, chronic health difficulties such as diabetes and pain and substance addictions. Contextual behavioural approaches aim to increase psychological flexibility of participants via increases in the use of acceptance and mindfulness strategies, thereby assisting them to deal with their cognitions and emotions in more helpful ways resulting in reductions in physical and mental health difficulties and other psycho-social conditions (Hayes, Luoma, Bond, Masuda, & Lillis, 2006). Some researchers have suggested that these approaches may extend the benefits of traditional Social Learning Theory based parenting interventions (Dumas, 2005; Greco & Eifert, 2004) by assisting parents to develop

strategies for dealing with thoughts and emotions that may act as barriers to effective parenting during times of distress. The approach has only begun to be used in the areas of parenting and trauma, however, the objectives of the approach and the targets of change are consistent with Kazak's PMTSM in that they aim to target an individual's appraisal of their thoughts and emotions, and may therefore help parents to reduce the level of distress they experience in relation to the events surrounding their child's illness/injury.

Program Adherence

The parent intervention groups will be facilitated by five mental health professionals (e.g., psychologists and therapists). The intervention is highly manualised and includes verbatim examples instructing facilitators on how to introduce key concepts during each session. Each session will contain a schedule outlining the strategies that must be covered during the session. To further ensure treatment fidelity the following strategies will be employed:

- Each session will contain an adherence checklist and facilitators are responsible for adhering to the checklist and completing it after each session.
- All facilitators will undergo extensive training in the delivery of the intervention. The training will include observing the sessions being delivered by the leading clinician, role playing session content and receiving feedback regarding the delivery of session content.
- Audio of the sessions will be recorded using the Google Hangouts program. The audio files will be stored on a password and firewall protected computer (on the hard drive) which can only be accessed by members of the research team. The taped sessions will be used to evaluate how the facilitators' perform in the session – e.g., their adherence to the program material, their clinical delivery skills etc. A team member will code aspects of the program to ensure treatment fidelity and to give an indication of inter-rater reliability.

Treatment Arms

1. Take A Breath (TAB)

TAB will aim to provide parents with practical strategies and approaches to better cope with the traumatic experience of having a seriously ill/injured child. The intervention will incorporate strategies that seek to directly target the parents' perceptions of the events surrounding their child's illness. The intervention will use a range of metaphors and experiential activities based on the evidence-based approaches described above, to assist parents to explore their current context and how best to cope as an individual, a parent and a family during and beyond their child's diagnosis, treatment and recovery. Participants will be encouraged to practice ideas and strategies from the program in between each session. Each session will involve reflection of past session content and parent attempts to implement program strategies.

TAB will incorporate strategies that assist parents to:

- Deal with the negative thoughts and emotions (stress, worry, anger, sadness) that result from having a child with a SCII
- Maintain their parenting role in the face of their child's SCII and their own emotional responses
- Develop plans for handling the disruptions to family relationships, roles and activities
- Maintain/develop supportive social connections/networks

2. Waitlist control
   The waitlist control group will receive standard clinical care within the hospital, including access to nurses, consultants, social workers, and psychologists. After completion of the pre and post intervention questionnaire as the comparison group, the waitlist group will then be offered the TAB intervention if they are interested.

6. SUBJECT ENROLLMENT FOR SCREENING OF PARENTS

6.1 Recruitment

The details of the project and the study objectives will be provided to key staff from the RCH Departments via a face to face briefing. These staff will be asked to promote the study and the parent program to new families (within four weeks of diagnosis) attending the department for treatment. A screening process will be used to determine eligibility for the parent intervention program.

Participants will be parents of children during their first presentation for a diagnosis of cancer, cardiac disease, or who have been admitted to hospital in the PICU department. Participants will primarily be parents of children receiving inpatient services, however, in some instances parents may be seeking services for their child on an outpatient basis. Participants will be asked to consent to participate in Screening in order to determine their eligibility into the RCT trial (parent intervention groups), to allow the research team to access their child's medical records for relevant medical and demographic information, and to consent to be recontacted in 3-6 months' time to be invited to participate in the RCT.

The Screening Questionnaire involves the completion of the ASDS on a single occasion within four weeks of their child's diagnosis (T1) along with the Demographic Questions. Parents who report pre-existing psychological conditions and/or other trauma/death will be considered ineligible for the RCT. The information obtained from the Screening Questionnaire, specifically the results from the ASDS, will provide key inclusion criteria regarding eligibility into the RCT. Parents **must score greater than or equal to 9 on items 1 to 5 (dissociation items) AND greater than or equal to 28 on items 6 through to 19 (combined items from hypervigilance, re-experiencing and avoidance subscales)** using the cluster scoring method. The ASDS will also provide important acute distress data on parents psychosocial functioning within four weeks of their child's diagnosis/surgery/admission and the demographic information gathered will ask about marital status, employment etc.

Eligibility Criteria

Inclusion Criteria

To be eligible to participate parents must meet all of the following criteria:

1. Parents must have a child aged between zero and eighteen years during their first presentation treatment for a SCII (cancer, cardiac disease or who have been admitted to hospital in the PICU department)
   - Cardiac – child required surgery within the 1st month of life. This decision was made following consultation with the Head of Cardiology who recommended that we target this group of parents as they are more likely to be traumatised compared to other parents of children with cardiac disease.
   - Cancer – all cancer types
   - PICU – child's stay at PICU must be greater than or equal to 48 hours
2. Parents may be male or female

3. Both mothers and fathers can participate together or individually, however the eligible parent must take part
4. Parents must have an active and current parenting role with the child (defined as the person(s) who perceives that they have an active parenting role)
5. Parents must be able to comply with the study intervention and assessment protocols. This will be determined by the researcher during registration contact with the parent
6. Parents may have multiple children diagnosed with an SCII
7. Parents must be over 18 years

6.2.2 Exclusion Criteria

1. Parent has experienced other major trauma (e.g., death of child, partner or other loved one in two months prior to child's diagnosis)
2. Parent has a pre-existing psychological condition
3. Parent does not have current access to children
4. Parent has limited spoken English and/or literacy. This will be determined by the researcher during initial consultations with the parent
5. Child is not expected to live longer than 6 months

SUBJECT ENROLLMENT AND RANDOMISATION FOR RCT

Recruitment

Families determined as eligible from the Screening questionnaire (ASDS cluster scoring: a score of 9 or above on items 1 to 5 AND a score of 28 or above on items 6 to 19) will be invited to participate in the parent intervention program.

a. During the telephone call to invite parents to take part in the RCT the researcher will describe in full the requirements of participation in the efficacy trial. Parents will be sent a plain language statement and consent form (see Appendix 4: PICF – Parent Intervention Group – Parent Version) and a flyer detailing information about the TAB program. Parents will also be given the opportunity to ask questions. Parents will be asked if they would prefer the PICF to be sent via the mail or online. For those parents who have chosen to receive the PICF online, they will be provided with the option of having a copy of the PICF either mailed or emailed to them.
b. If neither parent is interested at that time, contact will cease unless the family indicates they would like time to consider the study.
c. If a parent is interested in participating they will be asked to sign and return the consent form to the Research Team in the provided reply paid envelope. For those parents who have selected to consent online, they can either return the signed PICF via email Parent and Researcher consent may therefore not be dated the same.
d. Upon receipt of signed consent forms, parents will be enrolled into the study and then randomly allocated to one of the two treatment arms.
e. If a family verbally consents to continue their participation when called, this verbal consent will be noted in the trial database. At this point, the parents will be enrolled into the study and then randomly allocated to one of the two treatment arms. The researcher will again encourage the participating parent to

return written or online consent. If a parent requires the consent form to be mailed out again, or if an email consent form is preferred, then the form will be sent to the family once more, in another attempt to obtain written consent. If we again have difficulty obtaining formal written or online consent, a second verbal consent will be obtained over the phone prior to questionnaires being sent out and participation in the program commences. During this same phone call, a member of the research team will determine days and times that best suit the parent for attending a parent program and schedule them into a group. Parents allocated to the W-L arm will be advised when to expect the first set of questionnaires and advised of the approximate starting month for their participation in a group (following the completion of their post questionnaire).

f. Pre-assessment measures will be distributed two weeks prior to the commencement of TAB group sessions to parents enrolled in the two treatment arms. Pre-assessment measures must be completed by parents prior to the commencement of the parent program. Parents have the choice to complete the questionnaires online or via paper/pen format.
g. Any pre-assessment measures not returned/completed prior to the commencement of the parent program will be followed up. Parents allocated to the W-L arm will be followed up individually via their preferred method of contact. For those parents within the W-L arm that have failed to return written consent forms, another consent form will be sent with these pre-questionnaires, and they will be encouraged to return the signed consent forms when they return the pre-assessment measures.

Parents may choose to withdraw permission to participate at any time and request removal of their information from the database.

Randomisation Procedures

Eligible parents will be randomised to either the parent intervention group or the W-L group. This will typically occur between three and four months post their child's diagnosis.

Three randomisation lists will be generated, one list per participating department. This will enable separate analysis by illness group as well as an overall exploration of outcomes for families of children with SCII. Each randomisation list will contain twenty six spaces. Participants will be randomly allocated to one of the two treatment arms using The RCH Department, Clinical Epidemiology and Biostatistics Unit (CEBU), computerised randomisation plan generator. This program will randomise each participant to one of the two treatment arms using the method of randomly permuted blocks. The end result will be two randomly allocated treatment arms of equal size divided between three randomisation lists organised according to Hospital Department (Cancer, Cardiac and PICU).

7.4 Blinding Arrangements

Research team members responsible for recruiting families to the study or delivering the TAB intervention to families will be blind to the randomisation list. No researcher involved with recruitment or clinical delivery will have access to the randomisation list, thereby ensuring they are blind to client allocation and ensuring the randomness of allocation. A research officer, not involved in either the recruitment process or clinical delivery, will manage the randomisation list. When a consent form is received the research assistant responsible for recruitment will contact the research officer to have the participant placed on the randomisation list for the correct department using their subject identification code.

The research assistant will be informed of the allocation and will then contact the parent to discuss the allocation and plan their enrolment in a group as described above.

Researchers conducting the data analysis will be blind to participant identity and allocation. Analysis will be conducted on re-identifiable data only. A research assistant not responsible for analysis will conduct a re-identification of participant if needed.

Participants will be partially blinded. That means that participating parents will know if they have been randomised to W-L or the parent intervention arm (TAB) once they have been advised whether they will receive the intervention immediately or in two-six months' time.

7.5 Subject Withdrawal

7.5.1 Reasons for withdrawal

Participants are free to withdraw from the study at any time upon their request or the request of their legally acceptable representative.

The Research Team may withdraw a participant from the study (parent intervention and follow up procedures) if:

- During the course of the study, one of the exclusion criteria is met (e.g., the ill/injured child dies)
- The participant experiences a serious or intolerable adverse event
- Early discontinuation is required for any reason

The Researchers will also withdraw all participants from the study if the study is terminated.

7.5.2 Handling of withdrawals and losses to follow-up

When a participant withdraws from the study, the participant will be contacted by a RT member to discuss their reason/s for withdrawal if they have not already informed the research team. The interviewer will open conversation regarding withdrawal or non-participating by generally asking participants about their reasons for research discontinuation. Participants can choose not to take part in the call. Once a participant has withdrawn they are not required to complete any follow-up assessments and participation in the study is discontinued. Any information gathered during the course of the study from withdrawn participants will be kept unless participants request that their information be destroyed.

If RT members are concerned about the wellbeing (e.g., elevated scores on psychometric measures) of withdrawn participants actions to ensure the safety and wellbeing of these participants will be taken (e.g., referral information will be provided, families encouraged to link in with their nurse coordinator or social worker).

7.6 Trial Closure

Once the designated number of participants in the study has been reached (e.g., 184 participants have been randomised the RT will inform The RCH departmental staff who have been promoting the study, that the study is closed to new participants. If potential participants contact the RT to participate, they will be provided with appropriate alternative referral options if needed. As outlined in the PICF, participants will be provided with a summary of group outcomes of the study. It is not anticipated that the study will cease prematurely. The only foreseeable circumstance would be if funding for the study was withdrawn. However in this case the RT would seek additional funding to complete the study. Should any difficulties arise with recruiting the required number of participants, an extension to continue the study will be sought. Again additional funding will be sought to extend the study to ensure that the adequate number of participants has been achieved to complete the study.

8. STUDY IMPLEMENTATION SCHEDULE

8.1 Screening

A maximum of 788 parents will participate in the screening process allowing for an uptake rate of seventy per cent of these eligible parents (n = 552). Parents will be provided with the Screening Questionnaire within four weeks of their child's diagnosis.

8.2 Parent Intervention Groups

A total of 263 parents will participate in the main RCT study. It is anticipated that up to 30 parent groups will be delivered. A maximum of 10 parents will be enrolled into each group to ensure that parents can participate fully in all aspects of the program.

Parent groups will be scheduled on different days and times, with evenings and/or weekends being available. Sessions will be held once per week for a total of five consecutive weeks with a sixth session held two to four weeks later. The sixth session is included as it provides parents with a short period to practice and implement the skills and ideas taught during the program and to address any difficulties or barriers that arise when implementing them. All sessions will be delivered via online videoconferencing using Google Hangouts.

Parent programs will be commenced when enough parents have been randomised to the TAB treatment arm to constitute a group (a minimum of 5). Parents randomised to the Wait List arm will receive their pre questionnaires at the same time as the parents scheduled to commence that TAB group. In this way the timing for their assessment points will be matched to the participants in the TAB treatment arm.

Parents will be monitored by the practitioner facilitating the group they are attending throughout the intervention and at each assessment point. Evaluation questionnaires will be used to support the clinical judgment of the practitioner.

8.3 Evaluation

Assessment data will be collected from all participating parents. Additionally, data regarding the child's diagnosis and treatment will be extracted from the child's medical file.

Parents

Parents will be asked to complete a series of participant measures on two separate occasions. The participant measures from the TAB arm will then be compared to the participant measures from the W-L at two time points (See Table 2 for detail).

- T2: Two weeks prior to parents attending the parent program (Pre)
- T4: Immediately following completion of the parent program (Post)

Immediately after T4 it is anticipated that parents in the W-L arm who wish to participate will have had the opportunity to register and/or participate in the TAB parent group.

It is anticipated that completion of the questionnaire package will take parents approximately twenty to thirty minutes at each data collection time point. Parents will be provided with the questionnaire and an envelope for sealing and returning the completed questionnaire to the research team. Parents will also be provided with the option of completing the questionnaires online.

			Parent
		Week	***Task/Assessment Category***
**Time Point**	***T1: Screening***	***1***	Demographic questions<br>Acute Stress Disorder Scale (not for the developmental disability pilot study)
	T2: Pre-Assessment	***20***	Parent posttraumatic stress<br>Experience of illness<br>Child wellbeing<br>Child Psychopathology<br>Parent wellbeing<br>Parent Psychopathology<br>General Family Functioning
	T3: Intervention	***23***<br>***24***<br>***25***<br>***26***<br>***30***	Parent Program – Session 1<br>Parent Program – Session 2<br>Parent Program – Session 3<br>Parent Program – Session 4<br>Parent Program – Session 5<br>Parent Program – Session 6
	T4:Post Intervention	***30***	Parent posttraumatic stress<br>Experience of illness<br>Child wellbeing<br>Child Psychopathology<br>Parent wellbeing<br>Parent Psychopathology<br>General Family Functioning<br>Acceptability
	T5: 6-month Follow-up	***58***	Same as pre-assessment measures only for intervention families

Table 2. Implementation Schedule

Screening Questionnaire

Acute Stress Disorder Scale (ASDS; R. A. Bryant, Moulds, & Guthrie, 2000)

The ASDS is a 19-item self-report measure designed to assess acute stress disorders in individuals in the acute period (up to 4 weeks) following a traumatic event and who may be at risk of developing PTSD. The ASDS indexes acute stress disorder based upon the criteria of the Diagnostic and Statistical Manual of Mental Disorders 4th ed. (DSM- IV). The ASDS measures 4 cluster of symptoms - dissociation (5 items), re-experiencing (4 items), avoidance (4 items) and arousal (6 items). Responses are measured on a 4-point scale ranging from Not at All to Very Much. Internal consistency for the total scale was reported to be .96 and .84 for dissociation, .87 for experiencing, .92 for avoidance and .93 for arousal.

Demographic Questions

A series of questions have been developed to obtain relevant demographic information from participating parents. These were developed in consultation with the chief investigators on the team, drawing on their research and clinical experience. A total of

19 items are to be completed by parents at Phase One Screening only and ask about marital status, employment status, and education completed, language(s) spoken at home.

Pre-Assessment Parent Intervention Group Questionnaire

Primary Outcome Measure – Posttraumatic stress

Posttraumatic Stress Disorder Checklist – Version 5 (PCL5; Blevins et al., 2015)

The PCL5 is a 20 item self-report instrument used to measure posttraumatic stress symptoms in parents. The 20 items assess the 20 DSM-V PTSD criteria. Parents were asked to complete the measure in relation to their child's diagnosis. The Total Score (range = 0-80) was used, with higher scores indicating greater PTSS. Internal consistency for the Total Score in the current study was α=0.93.

Secondary Outcomes

Experience of Illness

Family Management Measure (FaMM; Knafl et al., 2009): measure taken from http://nursing.unc.edu/research/famm/*)*

The 45 item (53 if participant has a partner) FaMM was developed to measure how families manage caring for a child with a chronic condition/illness and the extent to which they incorporate condition management into everyday family life. There are five summated scales for all parents measuring the dimensions of Child's Daily Life, Condition Management Ability, Condition Management Effort, Family Life Difficulty, and View of Condition Impact as well as a sixth scale only for partnered parents measuring the dimension of Parental Mutuality. Due to overlap with other questionnaires in the protocol, the Parent Mutuality and Condition Management Ability are only included. Items are scored from 1 to 5, meaning strongly disagree to strongly agree. Higher scores on two of the scales (Condition Management Ability, and Parental Mutuality) indicate greater ease in managing the child's condition. Higher scores on the other two scales (Condition Management Effort, and Family Life Difficulty) indicate greater difficulty in managing the condition. Internal consistency reliability (ICR) for the scales, adjusted for inter-parental correlation, ranged from .72 to .90 for mothers and .73 to .91 for fathers (Knafl, G., et al., unpublished manuscript). Test-retest reliability was based on responses from 65 parents retested within 2-4 weeks and adjusted for inter-parental correlation. It ranged from .71 to .94.

Parent Experience of Child Illness (PECI; Bonner et al., 2006)

The PECI is a 25 item measure of parent adjustment to a child's serious or chronic illness. Four factors found: Guilt and Worry, Emotional Resources, Unresolved Sorrow and Anger, and Long-term Uncertainty. Bonner and colleagues (2008) (Bonner, Hardy, Willard, Hutchinson, & Guill, 2008) reported test-retest reliability correlation coefficients of .83 to .86. Internal reliability alphas were between .74 and .85. They also found adequate discriminant and convergent validity. They also found the PECI to demonstrate sensitivity to differences in participant's medical status. These results

were found in a population of paediatric cancer patients. Further testing across other medical groups would need to be conducted.

Child Wellbeing

Paediatrics Quality of Life (PedsQL; Varni, Katz, Seid, Quiggins, & Friedman-Bender, 1998).

The PedsQOL is a brief 23 item measure which assesses health-related quality of life in children and adolescents across four dimensions. These dimensions, as delineated by the World Health Organisation include Physical, Emotional, and Social and School functioning. A Total Score, a Physical Health Summary Score and Psychosocial Health Summary Score are derived. The inventory takes approximately four minutes to complete. The inventory has a child self-report and parallel parent proxy report format for ages 5-7, 8-12, and 13-18 years, which can be self-administered except ages 5-7 which has an interview format. Items are scored on a five-point Likert scale, ranging from 'never a problem' to 'almost always a problem.' The child self-report for ages 5-7 is simplified for a three-point Likert scale. An example item from the child report is "It is hard for me to walk more than one block". A higher PedsQL score indicates a better quality of life. Internal consistency for the Total Scale Score (a = 0.88 child, 0.90 parent report), Physical Health Summary Score (a = 0.80 child, 0.88 parent), and Psychosocial Health Summary Score (a = 0.83 child, 0.86 parent) have been reported to be acceptable ((Varni et al., 1998). Reported to be responsive to clinical change over time. The PedsQL distinguished between healthy children and paediatric patients with acute or chronic health conditions.

9.3.3 Child Psychopathology

The Brief Infant Toddler Social Emotional Assessment (BITSEA; Briggs-Gowan & Carter, 2002).

The BITSEA will be used as a measure of parent perceptions of their infant or child's difficult behaviours and social-emotional problems. This is a questionnaire for infants that are between 12 and 36 months old, and is therefore an alternative to the SDQ for the infant cardiology group. The BITSEA consists of 42 items, which fall into seven domains: internalizing, externalizing, dysregulation, competence, social relatedness, maladaptive, and atypical. Items are rated on a 3-point scale (0=not true/rarely, 1=somewhat true/sometimes, 2=very true/often), with a higher score suggesting better functioning. An example of an item is "Your child: Hits, bites or kicks you". The BITSEA has good internal consistency and inter-rater reliability, and excellent test-retest reliability (Briggs-Gowan, Carter, Irwin, Wachtel, & Cicchetti, 2004). Internalising, externalising and dysregulation subscales will only be administered.

OR (depending on age of child)

Behavior Assessment System for Children, Second Edition (BASC-2; Reynolds & Kamphaus, 2004)

The BASC-2 measures maladaptive and adaptive behaviours and self-perceptions of children. The parent rating scales were utilized according to the child's age, and

consist of 134-items for children aged 2-5 years, and 160-items for children aged over 6 years. Respondents rate how the child has behaved in the last several months (e.g. ‘is easily upset’; ‘has trouble making new friends’), on a 4-point scale from “never” to “almost always”. The BASC-2 has nine clinical scales, including Hyperactivity, Aggression, Conduct Problems, Anxiety, Depression, Somatization, ATypicality, Withdrawal, and Attention Problems, and three adaptive scales including Adaptability, Social Skills, and Leadership. There are also four composite scores including Externalizing Problems, Internalizing Problems, Behavioral Symptoms Index, and Adaptive Skills. The BASC-2 has good psychometric properties, with established validity, and internal consistency estimates ranging from the middle .80s to middle .90s (Tan, 2007).

Parent Wellbeing

Parental Psychological Flexibility Questionnaire- Short Form (PPF- SF; Burke, 2009)

The PPF- short form (Burke, 2009) is a 19 item instrument that measures parental psychological flexibility. The PPF consists of three subscales designed to measure key elements of psychological flexibility: Emotional Willingness, Cognitive Defusion and Acceptance and also provides an overall level of parental psychological flexibility via a Total Score. The PPF was developed in Study 1 of the thesis that the current study is part of. The measure demonstrates adequate psychometric properties, with good validity and reliability for the Total Scale (α =.89) and each of the subscales, Cognitive Fusion (α =.90), emotional Willingness (α = .74) and Acceptance (α = .79). An example item is “My emotions get in the way of being the type of parent I would like to be”. The items are measured on a 7-point Likert scale from 1 = Never true to 7 = always true.

Five Facet Mindfulness Questionnaire – Short Form (FFMQ-SF; Bohlmeijer, ten Klooster, Fledderus, Veehof, & R., 2011)

The FFMQ - SF, consisting of 24 items, is a multifaceted measure of a general tendency towards day to day mindfulness. The five facets include Observing, Describing, Acting, Nonjudging of Inner Experience, and Nonreactivity to Inner Experience. Items are rated on a 5-point Likert scale ranging from 1 (never or very rarely true) to 5 (very often or always true). For all facets, higher scores reflect higher levels of mindfulness. The five facets demonstrated adequate to good internal consistency, with alpha coefficients ranging from .75-.87 (Baer et al, 2006).

Valuing Questionnaire (VQ; Smout, Davies, Burns, & Christie, 2011).

The VQ8 is an 8-item questionnaire assessing the degree to which people live by their values and is broken down into two subscales – Progress (extent to which people felt that they lived their values in the past week) and Obstructed (the extent to which cognitive and emotional barriers interfered with acting out their values in the past week). Respondents answer items on 6 point likert scale from 0 – *not true at all* to 6 – *completely true*. The VQ8 has very good internal consistency for both factors – Progress α= 0.90 and Obstructed α= 0.83

Acceptance and Action Questionnaire – II (Bond et al., in press).

The AAQ-II, containing 7 items, assesses psychological flexibility/inflexibility (e.g., acceptance and experiential avoidance) on a 7 point likert scale ranging from 1 (never true) to 7 (always true). Higher scores reflect greater experiential avoidance and immobility and lower scores are indicative of greater action and acceptance. Psychometric testing reported mean alpha coefficient across the six samples tested of .84 (ranging between.78 - .88), and the 3- and 12-month test-retest reliability is .81 and .79, respectively (Bond et al., in press). Findings from these studies indicate that the measure is related to variables to which it is theoretically tied to. For example, higher levels of psychological inflexibility, as measured by the AAQ-II, are associated with greater levels of anxiety, stress, depression as well as overall psychological distress.

Life Events Questions

The life events questionnaire was developed by the senior investigators to obtain information about potential psychosocial stressors that participating parents may have experienced in the past 12 months (in addition to their child's serious illness/injury). The questionnaire contains a total of 14 items and asks about job loss and reduced work hours, recent pregnancies, moving home, suffering a serious illness/injury themselves, separation/divorce, or whether they have experienced an event they found traumatic. The parent is also asked to report on their partner. In addition to these items, a final 15th item was included to ask about a history of mental illness in the year prior to their child's illness/injury.

Parent Psychopathology

The Depression Anxiety Stress Scale, (P. Lovibond & S. Lovibond, 1995) was included as a measure of the symptoms of depression, anxiety and stress. This 21-item factor requires respondents to indicate how much each item applies to them on a scale of '0 Did not apply to me at all' to '3 Applied to me very much or most of the time. The DASS-21 has been shown to have good internal consistency reliability, with Cronbach's alpha coefficients reported as .88 for the Depression Scale, .82 for the Anxiety Scale, .90 for the Stress Scale, and .93 for the Total Scale (Henry, & Crawford, 2005).The DASS will also be administered at pre, post and follow up one and two.

Acceptability

Consumer Satisfaction Scale (Parenting Research Centre, 2010).

The CSS is a measure of consumer satisfaction with parent training programs. It assesses the quality of the service provided; how well the program met the parent's needs and changed behaviour, and whether the parent would recommend the program to others. Parents are also prompted to make general comments or suggestions about the program. The instrument is included as a measure of parental perceptions of the program, the information provided by parents will be used to refine and improve the program. This measure will be completed at Time 4 (post program completion) only.

11. STATISTICAL METHODS

Sample Size Estimation

In order to detect a difference of 6.0 (SD: 13.9) points (thought to be a clinically important difference in parents' experience of distress) in the Posttraumatic Stress Disorder Checklist (PCL- 5) measure, between the two treatment arms, with a significance level of 0.05 and power of 0.80, 82 participants will be required per arm. Allowing attrition, we therefore aim to recruit a total of 184

11.2 Population to be analysed

Data will be cleaned in the Statistical Package for the Social Sciences (SPSS) using procedures outlined in Tabachnick and Fidell (2007). Missing value analyses will be conducted and expectation maximisation techniques will be used to impute subscale data missing completely at random. Data will be imputed for participants completing at least 70% of items on that subscale; all other items on the subscale will be used to estimate missing data. If participants completed less than 70% of a subscale their data will be not imputed and the participant will not be included in analysis involving that subscale.

Both completer and intention-to-treat analyses will be conducted. Average closest match techniques (Elliot & Hawthorne, 2005) will be used to replace missing post and follow-up data for those who did not return questionnaires. This involves using the average post/follow-up-intervention value obtained by participants reporting the same pre-intervention score (or the four participants reporting the closest pre-intervention score), to replace the missing data for that case. This technique is considered a reliable and efficacious approach for managing missing data (Elliot & Hawthorne, 2005).

11.3 Statistical Analysis Plan

Demographic, illness group (oncology, PICU, cardiac) and screener characteristics will be compared between groups. For parent-level data (for couples that were both eligible), categorical variables will be compared using Generalised Estimating Equation (GEE) models to account for within-couple correlation (with the exception of parent sex and illness group). A GEE with a Gaussian distribution will be employed to compare group means of the screening measure. Time between measurements using linear regression models, and illness group distributions will be compared using a chi-squared test.

GEE models will compare mean outcome measures between groups at T3, and GEE models will also account for within-couple correlations. Effect size Cohen's d will be calculated, and interpreted as small<0.2, medium>0.2-0.5, large=>0.5-0.8, very large >0.8 (Cohen, 1988).

Further analyses will include exploration of the predictors of intervention outcome, and a thorough analysis of treatment adherence, compliance with intervention and other process variables. Detailed descriptive analysis of pre-intervention child, parent and family characteristics will also be conducted to thoroughly explore the characteristics of families attending a parent focused, psychosocial intervention.

11.4 Interim Analyses

No interim analyses will be done for this study.

12. DATA MANAGEMENT

12.1 Data Collection and Data Storage

Participant questionnaire results (hereafter defined as source data) will be collected using paper pencil format or online across the T2 and T4 points. Primarily these questionnaires are completed online using the RedCap program as parents prefer this format. Participants completing them using paper and pencil will be provided with a reply paid envelope to seal their results and return it to the RT at the MCRI. A research assistant will check the incoming mail on a daily basis. When the research assistant receives the completed questionnaire he/she will tear the first page off the questionnaire to re-identify and record the participant's code on the questionnaire on each remaining page.

The source data will be managed using the RedCAP database, and will be held at the MCRI in a secure room on a password protected computer. The RedCAP Database will be used to track participants' movement through the study, including eligibility, dropout and data collection processes SPSS will be used to store and analyse the re-identifiable data for participants. Hard copy consent forms and questionnaires will be kept separately in a locked compactus at the MCRI. The guidelines contained in the Consolidated Standards of Reporting Trials (CONSORT) statement will be adhered to. This enables detailed recording of participants across time. As such, information will be collected on both eligible and ineligible families, as well as families that refuse to participate. For ineligible and refusals, information will track demographic information (SES, postcode, age of child, family structure) and reasons for ineligibility/refusal. No identifying information will be collected on these families. Participants that withdraw from the study after commencement will be phoned, with their permission, to have a short conversation about their main reason from withdrawing from the project. The information will help to track reasons for participant retention and loss to the program and/or study.

Details regarding the patients' illness including date of diagnosis, diagnosis type, number of visits to the Emergency Department (ED) and number of days of admission will also be collected for study participants (parents who have returned consent forms). This information will be obtained from departmental/hospital databases by requesting a medical record extraction from The RCH Patient Information System (IBA). Patient diagnoses will be described. Length of treatment will be calculated from date of diagnosis to date for parents' completion of questionnaires. A member of the RT will collect the record and store it in a secure filing cabinet at MCRI prior to returning it to medical records.

All audio files recorded during the delivery of the intervention will be stored on a password and firewall protected computer in a locked room and will contain audio from both practitioner and parents. They will also be securely shared with the team's clinical supervisor using the MCRI OwnCloud system.

13.3 Registration as Clinical Trial

The project has been registered as a clinical trial with the Australian New Zealand Clinical Trials Registry (ANZCTR) which sets the standards for the uniform reporting of the minimum registration data set as determined by the World Health Organization and the International Committee of Medical Journal Editors. ANZCTR Registration Number is ACTRN12611000090910.

15. REFERENCES

Barak, A., Hen, L., Boniel-Nissim, M., & Shapira, N. a. (2008). A Comprehensive Review and a Meta-Analysis of the Effectiveness of Internet-Based Psychotherapeutic Interventions. *Journal of Technology in Human Services, 26*(2-4), 109-160. doi: 10.1080/15228830802094429

Best, M., Streisand, R., Catania, L., & Kazak, A. E. (2001). Parental distress during pediatric leukemia and Posttraumatic Stress Symptoms (PTSS) after treatment ends. *Journal Of Pediatric Psychology, 26*(5), 299-307.

Blevins CA, Weathers FW, Davis MT, Witte TK, Domino JL. (2015). The Posttraumatic Stress Disorder Checklist for DSM-5 (PCL-5): Development and Initial Psychometric Evaluation. *Journal of Traumatic Stress.* 28, 489-498.

Bohlmeijer, E., ten Klooster, P., Fledderus, M., Veehof, M., & R., B. (2011). Psychometric Properties of the Five Facet Mindfulness Questionnaire in Depressed Adults and Development of a Short Form. *Assessment, 18*, 308-320.

Bonner, M. J., Hardy, K., Guill, A., McLaughlin, C., Schweitzer, H., & Carter, K. (2006). Development and validation of tthe parent Expereince of Child Illness. *Journal of Pediatric Psychology, 31*, 310-321.

Bonner, M. J., Hardy, K., Willard, V., Hutchinson, K., & Guill, A. (2008). Further validation of the Parent Experience of Child Illness Scale. *Children's Health Care, 37*, 145-157.

Briggs-Gowan, M., & Carter, A. (2002). Brief infant-toddler social and emotional assessment (BITSEA) manual, version 2.0. In Y. University (Ed.). New Haven.

Briggs-Gowan, M., Carter, A., Irwin, J., Wachtel, K., & Cicchetti, D. (2004). The Brief Infant-Toddler Social and Emotional Assessment: Screening for social-emotional problems and delays in competence. *Journal of Pediatric Psychology, 29*(2), 143-155.

Bryant, R. A., Moulds, M. L., & Guthrie, R. M. (2000). Acute Stress Disorder Scale: a self-report measure of acute stress disorder. *Psychological Assessment, 12*(1), 61-68.

Burke, K. (2009). *Parental Psychological Flexibility Questionnaire*.

Caplan, R., Siddarth, P., Gurbani, S., Hanson, R., Sankar, R., & Shields, D. (2005). Depression and anxiety disorders in pediatric epilepsy. *Epilepsia, 46*(5), 10.

Chesire, A., Barlow, J., & Powell, L. (2010). The psychosocial well-being of parents of children with cerebral palsy: A comparison study. *Disability Rehabilitation, 32*(20), 1673-1677.

Cleveland, L. (2008). Parenting in the neonatal intensive care unit. *Journal of Obstetric, Gynecologic, & Neonatal Nursing, 37*, 666 - 691.

Cook, J., & Doyle, C. (2002). Working alliance in online therapy as compared to face-to-face therapy: Preliminary results. . *Cyberpsychology and Behavior, 5*, 95-105.

Dumas, J. (2005). Mindfulness-based parent training: Strategies to lessen the grip of automaticity in families with disruptive children. *Journal of Clinical Child and Adolescent Psychology, 34*(4), 779-791.

Elliot, P., & Hawthorne, G. (2005). Imputing missing repeated measures data: how should we proceed? *Australian and New Zealand Journal of Psychiatry, 39*, 575-582.

Feeney, T., & Ylviksaker, M. (1995). Choice and routine: Antecedent behavioral interventions for adolescents with severe traumatic brain injury. *The Journal of Head Trauma Rehabilitation, 10*, 67-86.

Greco, L., & Eifert, G. (2004). Treating parent-adolescent conflict: Is acceptance the missing link for an integrative family therapy? *Cognitive and Behavioral Practice, 11*(3), 305-314.

Hall, E., Saxe, G., Stoddard, F., Kaplow, J., Koenen, K., Chawla, N., . . . King, D. (2006). Posttraumatic stress symptoms in parents of children with acute burns. *Journal of Pediatric Psychology, 31*(4), 403 - 412.

Hayes, S., Luoma, J., Bond, F., Masuda, A., & Lillis, J. (2006). Acceptance and commitment therapy: Model, processes and outcomes. *Behavior Research and Therapy, 4*, 1-25.

Hudson, M., Mertens, A., Yasui, Y., Hobbie, W. L., Chen, H., Gurney, J., . . . Oeffinger, K. (2003). Health Status of Adult Long-term Survivors of Childhood Cancer: A Report From the Childhood Cancer Survivor Study. *Journal of the American Medical Association, 290*(12), 1583-1592.

Johnson, B., Franklin, L., Hall, K., & Prieto, L. (2000). Parent training through play: Parent-child interaction therapy with a hyperactive child. *The Family Journal: Counseling and Therapy for Couples and Families, 8*(2), 180-186.

Kazak, A., Alderfer, M., Streisand, R., Simms, S., Rourke, M., Barakat, L., . . . Cnaan, A. (2004). Treatment of posttraumatic stress symptoms in adolescent survivors of childhood cancer and their families: A randmized clinical trial. *Journal of Family Psychology, 18*(3), 493 - 504.

Kazak, A., & Barakat, L. (1997). Brief report: Parenting stress and quality of life during treatment for childhood leukemia predicts child and parent adjustment after treatment ends. *Journal Of Pediatric Psychology, 22*(5), 749 - 758.

Kazak, A., Rourke, M., Alderfer, M., Pai, A., Reilly, A., & Meadows, A. (2007). Evidence-based assessment, intervention and psychosocial care in pediatric oncology: A blueprint for comprehensive services across treatment. *Journal Of Pediatric Psychology, 32*(9), 1099 -1110.

Kazak, A., Simms, S., Barakat, L., Hobbie, W. L., Foley, B., Golomb, V., & Best, M. (1999). Surviving cancer competently intervention program (SCCIP): A cognitive-behavioural and family therapy intervention for adolescent survivors of childhood cancer and their families. *Family Processes, 38*, 176-191.

Kazak, A., Stuber, M., Barakat, L., Meeske, K., Guthrie, D., & Meadows, A. (1998). Predicting posstraumatic stress symptoms in mothers and fathers of survivors of childhood cancers. *Journal of the American Academy of Child & Adolescent Psychiatry, 37*(8), 823 - 831.

Knafl, K., Deatrick, J. A., Gallo, A., Dixon, J., Grey, M., Knafl, G., & O'Malley, J. (2009). Assessment of the Psychometric Properties of the Family Management Measure. *J. Pediatr. Psychol.*, jsp034. doi: 10.1093/jpepsy/jsp034

Landolt, M. A., Vollrath, M., Ribi, K., Gnehm, H. E., & Sennhauser, F. H. (2003). Incidence and associations of parental and child posttraumatic stress symptoms in pediatric patients. *Journal of Child Psychology and Psychiatry, 44*(8), 1199-1207.

Lewis, M., Hatton, C., Salas, I., Leake, B., & Chiofalo, N. (1991). Impact of the chidren's epilepsy program on parents. *Epilepsia, 32*(3), 365 - 374.

Lovibond, P. (1999). *Depression Anxiety Stress Scales (DASS)*. Taken from internet: http://www.psy.unsw.edu.au/dass/: (23.7.99).

Lovibond, P., & Lovibond, S. (1995). The structure of negative emotional states: Comparison of the Depression Anxiety Stress Scales (DASS) with the Beck Depression and Anxiety Inventory. *Behavior Research and Therapy, 33*(3), 335-343.

Mu, P.-F. (2005). Paternal reactions to a child with epilepsy: Uncertainty, coping strategies, and depression. *Journal of Advanced Nursing, 49*(4), 367 - 376.

Pai, A., Neff Greenley, R., Lewandowski, A., Drotar, D., Youngstrom, E., & Cant Peterson, C. (2007). A meta-analytic review of the influence of pediatric cancer on parent and family functioning. *Journal of Family Psychology, 21*(3), 407-415.

Pai, A., Patino-Fernandez, A. M., Mcsherry, M., Beele, D., Alderfer, M. A., Reilly, A. T., . . . Kazak, A. E. (2008). The Psychosocial Assessment Tool (PAT2.0): Psychometric properties of a screener for psychosocial distress in families of children newly diagnosed with cancer. *Journal Of Pediatric Psychology, 33*(1), 50-62.

Parenting Research Centre. (2010). *Consumer satisfaction scale*. Parenting Research Centre. Melbourne.

Reynolds, C. R., & Kamphaus, R. W. (2004). *Behavior assessment system for children (2nd ed.)*. MN: American Guidance Service.

Sahler, O., Varni, J., Fairclough, D., Butler, R., Noll, R., Dolgin, M., . . . Mulhern, R. (2002). Problem-solving skills training for mothers of children with newly diagnosed cancer: A randomized trial. *Developmental and Behavioral Pediatrics, 23*(2), 77 - 86.

Sanders, M., Markie-Dadds, C., & Turner, K. (2003). Theoretical, scientific and clinical foundations of the triple p-positive parenting program: A population approach to the promotion of parenting competence. *Parenting Research and Practice Monograph, 1*, 1-24.

Sawyer, M., Streiner, D., Antoniou, G., Toogood, I., & Rice, M. (1998). Influence of parental and family adjustment on the later psychological adjustment of children treated for cancer. *Journal of the American Academy of Child & Adolescent Psychiatry, 37*(8), 815-822.

Shaw, R., Deblois, T., Ikuta, L., Ginzburg, K., Fleisher, B., & Koopman, C. (2006). Acute stress disorder among parents of infants in the neonatal intensive care nursery. *Psychosomatics, 47*(3), 206 - 212.

Slifer, K., Tucker, C., Gerson, A., Cataldo, M., Sevier, R., Suter, A., & Kane, A. (1996). Operant conditioning for behavior management during posttraumatic amnesia in children and adolescents with brain injury. *The Journal of Head Trauma Rehabilitation, 11*, 39-50.

Smout, M., Davies, M., Burns, S., & Christie, A. (2011). *The valuing questionnaire* Manuscript in preparation.

Tabachnick, B., & Fidell, L. (2007). *Using multivariate statistics* (5th Ed. ed.). Boston: Allyn and Bacon.

Tan, C. S. (2007). Test Review: Reynolds, C. R., & Kamphaus, R. W. (2004). Behavior assessment system for children (2nd ed.). . *Assessment for Effective Intervention, 32*, 121-124.

Tyack, Z., & Ziviani, J. (2003). What influences the functional outcome of children at 6 months post-burn? *Brain, 29*, 433 - 444.

Varni, J. W., Katz, E. R., Seid, M., Quiggins, D. L. J., & Friedman-Bender, A. (1998). The pediatric cancer quality of life inventory-32 (PCQL-32). *Cancer, 82*, 1184 - 1196.

Webster-Stratton, C., & Hammond, M. (1997). Treating children with early-onset conduct problems: A comparison of child and parent training interventions. *Journal of Consulting and Clinical Psychology, 65*(1), 93-109.

Williams, J., Steel, C., Sharp, G., DelosReyes, E., Phillips, T., Bates, S., . . . Griebel, M. (2003). Parental anxiety and quality of life in children with epilepsy. *Epilepsy and Behavior, 4*, 483 - 486.

Zebrack, B., Zelter, L., Whitton, J., Mertens, A. O., L., Berkow, R., & Robison, L. (2002). Psychological outcomes in long-term survivors of childhood leukemia, Hodgkin’s disease, and mon-Hodgkin’s lymphoma: A report from the childhood cancer survivor study. *Pediatrics, 110*, 10.
