# Supplementary material for: Effect of a Videoconference-Based Online Group Intervention for Traumatic Stress in Parents of Children With Life-threatening Illness: A Randomized Clinical Trial
Source: JAMA Netw Open. 2020 Jul 31;3(7):e208507. doi: 10.1001/jamanetworkopen.2020.8507 (PMC7395233; doi:10.1001/jamanetworkopen.2020.8507)
Supplement: Supplement 2. — eTable 1. Take a Breath Session Content and Structure eTable 2. Comparison Between the Analytic Sample and Those That Consented but Were Lost/Dropped Out [file jamanetwopen-3-e208507-s002.pdf]

Supplementary Online Content

Muscara F, McCarthy MC, Rayner M. Effect of a videoconference-based online group intervention for traumatic stress in parents of children with life-threatening illness: a randomized clinical trial. *JAMA Netw Open*. 2020;3(7):e208507. doi:10.1001/jamanetworkopen.2020.8507

eTable 1. Take a Breath Session Content and Structure

eTable 2. Comparison Between the Analytic Sample and Those That Consented but Were Lost/Dropped Out

This supplementary material has been provided by the authors to give readers additional information about their work.

© 2020 Muscara F et al. *JAMA Network Open.*

eTable 1: Take a Breath Session Content and Structure

Session	Content
Session 1	Introductions<br>Orientation to technology<br>Group rules and process<br>Overview of program and introduction to BOLD analogy<br>Sharing stories<br>“B” – Breathe deeply and slow down<br>Mindfulness practice<br>Wrap up and set home practice
Session 2	Mindfulness practice<br>Home practice review<br>“O” – Observing feelings<br>Self-compassion<br>Wrap up and set home practice
Session 3	Mindfulness practice<br>Home practice review<br>“O” – Observing thoughts<br>Wrap up and set home practice
Session 4	Mindfulness practice<br>Home practice review<br>“L” – Listen to your values<br>“D” – Decide what matters and do it<br>Wrap up and set home practice
Session 5	Mindfulness practice<br>Home practice review<br>Group discussion and trouble shooting<br>Recap of program overview<br>Self as context<br>Wrap up and set home practice
3 Week Break	
Session 6 (Booster)	Mindfulness practice<br>Home practice review<br>Group discussion and trouble shooting<br>Functional behaviour change question<br>Recap of program overview<br>Wrap up and set home practice

© 2020 Muscara F et al. *JAMA Network Open.*

eTable 2: Comparison Between the Analytic Sample and Those That Consented but Were Lost/Dropped Out

	Lost/dropped out		Analysed		
	n	%	n	%	p*
PARENT	141		81		
Gender					.02
Male	48	34.0	16	19.8	
Female	93	66.0	65	80.2	
Marital status**					.67
Single	7	5.0	3	3.7	
Married	80	56.7	48	59.3	
Living with partner	27	19.1	11	13.6	
Missing	27	19.1	19	23.5	
Employment status**					.85
FT	37	26.2	18	22.2	
PT	17	12.1	13	16.0	
Casual	15	10.6	8	9.9	
Not employed	34	24.1	18	22.2	
Parental leave	5	3.5	4	4.9	
Missing	33	23.4	20	24.7	
Highest education**					.19
Postgraduate Degree	16	11.3	17	21.0	
Graduate Diploma/Certificate	7	5.0	7	8.6	
Bachelor Degree	31	22.0	16	19.8	
Diploma/Advanced Diploma	29	20.6	11	13.6	
Certificate	27	19.1	14	17.3	
Year 12	11	7.8	10	12.3	
Year 11 or below	19	13.5	6	7.4	
Missing	1	0.7	0	0.0	
ATSI**	5	3.5	1	1.2	.42
Missing	2	1.4	3	3.7	
LOTE at home	34	24.1	9	11.1	.02
Missing	2	1.4	0	0.0	
Aus. COB	103	73.0	64	79.0	.25

© 2020 Muscara F et al. *JAMA Network Open.*

Missing	0	0.0	1	1.2	
ASDS Screen	58.0	11.2	57.7	11.0	.56
Illness group					.15
Cardiology	36	25.5	24	29.6	
Oncology	43	30.5	32	39.5	
PICU	62	44.0	25	30.9	
CHILD	110		74		
Age @ dx [years], Mdn (IQR)***	1.6	0.8-5.1	1.5	0.4-6.9	.63
Illness group					.30
Cardiology	29	26.4	22	29.7	
Oncology	33	30.0	28	37.8	
PICU	48	43.6	24	32.4	

Mdn=median, IQR=interquartile range

*Missing excluded

**Fisher's exact

***Negative binomial distribution employed

© 2020 Muscara F et al. *JAMA Network Open.*
