# Supplementary material for: Effect of a Videoconference-Based Online Group Intervention for Traumatic Stress in Parents of Children With Life-threatening Illness: A Randomized Clinical Trial
Source: JAMA Netw Open. 2020 Jul 31;3(7):e208507. doi: 10.1001/jamanetworkopen.2020.8507 (PMC7395233; doi:10.1001/jamanetworkopen.2020.8507)
Supplement: Supplement 3. — Data Sharing Statement [file jamanetwopen-3-e208507-s003.pdf]

Data Sharing Statement

Data
Data available: Yes
Data types: Data dictionary, Deidentified participant data
How to access data: Available through the LifeCourse data registry at the Murdoch Children's Research Institute
When available: With publication

Supporting Documents
Document types: None

Additional Information
Who can access the data: Anyone requesting the data
Types of analyses: For specific purposes agreed by the authors
Mechanisms of data availability: With investigator support, following approval of a proposal
